# Silk: A Potential Medium for Tissue Engineering

**Published:** 2008-10-10

**Authors:** Cassandra Sobajo, Farhad Behzad, Xue-Feng Yuan, Ardeshir Bayat

**Affiliations:** ^a^ Plastic & Reconstructive Surgery Research, Manchester Interdisciplinary Biocentre, the University of Manchester, Manchester, M1 7DN, UK; ^b^ Biochemical Physics, Manchester Interdisciplinary Biocentre, The University of Manchester, Manchester, M1 7DN, UK

## Abstract

**Objective:** Human skin is a complex bilayered organ that serves as a protective barrier against the environment. The loss of integrity of skin by traumatic experiences such as burns and ulcers may result in considerable disability or ultimately death. Therefore, in skin injuries, adequate dermal substitutes are among primary care targets, aimed at replacing the structural and functional properties of native skin. To date, there are very few single application tissue-engineered dermal constructs fulfilling this criterion. Silk produced by the domestic silkworm, *Bombyx mori*, has a long history of use in medicine. It has recently been increasingly investigated as a promising biomaterial for dermal constructs. Silk contains 2 fibrous proteins, sericin and fibroin. Each one exhibits unique mechanical and biological properties. **Methods**: Comprehensive review of randomized-controlled trials investigating current dermal constructs and the structures and properties of silk-based constructs on wound healing. **Results**: This review revealed that silk-fibroin is regarded as the most promising biomaterial, providing options for the construction of tissue-engineered skin. **Conclusion**: The research available indicates that silk fibroin is a suitable biomaterial scaffold for the provision of adequate dermal constructs.

Human skin is a complex organ made of 2 layers of dermis and epidermis. The loss of the integrity of the protective barrier served by skin through injury or illness may result in infection, dehydration, and necrosis, which may ultimately lead to severe trauma and shock, with morbid consequences. Therefore, adequate cutaneous substitutes, which ideally, could replace all the structures and functions of native skin are essential to permit maximal recovery. Silk has recently been established as a biomaterial scaffold capable of fulfilling the properties required for effective cutaneous constructs.

## SKIN

Human skin anatomically and functionally consists of 2 primary layers:

1. The outer epidermis is composed of keratinized stratified squamous epithelium consisting mainly of keratinocytes.
2. The dermis is a dense irregular connective tissue primarily consisting of fibroblasts, which underlies and interdigitates with the epidermis.

**Figure 1:**
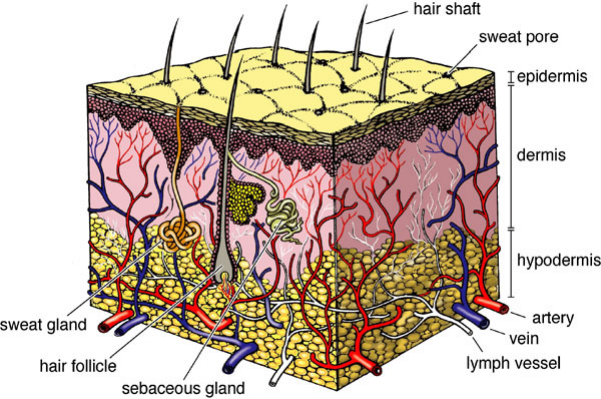
Normal human skin.

Underlying the dermis is the hypodermis (or subcutaneous layer), a loose connective tissue containing varying amounts of adipocytes (Figure 1). The margin between the dermis and the hypodermis is abrupt; however, the 2 regions are structurally and functionally well integrated through nerve and anatomizing vascular networks.

Skin has a diverse range of functions. It serves as a barrier against microorganisms and other environmental insults. It provides mechanical support against injury and radiation such as ultraviolet light. Also, skin protects the body against dehydration and provides sensory detection at the body surface.

## CUTANEOUS CONSTRUCTS

**Figure 2:**
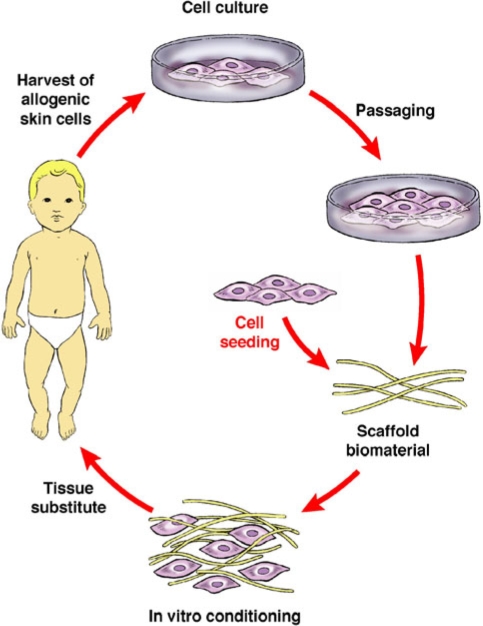
The basic elements of tissue engineering.

The development of cutaneoussubstitutes/constructs by tissue engineering (TE) has evolved from simple cultured autologous epidermal sheets to more complex bilayered cutaneous constructs (Figure 2). Currently, there are no tissue-engineered cutaneous constructs that can duplicate the complexity of the human skin. Mainly, a dermal construct is created with or without a temporary synthetic epidermis (Table 1).

## CULTURED CUTANEOUS CONSTRUCTS

**Figure 3:**
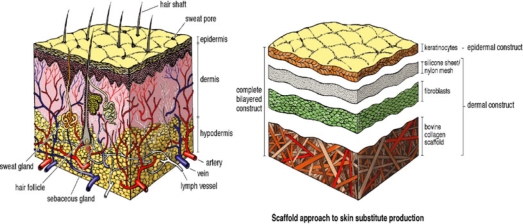
The scaffold approach to skin substitute production.

The attempts to create an effective skin substitute have been along the 3 TE approaches: (1) gel approach, (2) scaffold approach, and (3) self-assembly approach. The scaffold approach is the most commonly used in TE (Figure 3). It is used to create porous scaffolds, frequently from natural (eg, collagen) or biosynthetic biomaterials such as polyglycolic acid or polyvinyl alcohol. Classification of these scaffolds is based on the absence or presence of cellular components, to either acellular or cellular respectively. Both systems have been widely used in skin TE.

An in vivo study of cultured artificial dermal substitutes showed that an artificial dermis containing autologous cultured fibroblasts enhances the reepithelization of a full-thickness skin defect when compared to an acellular dermal substitute scaffold.^1^ This emphasized the significance of incorporating fibroblasts in all engineered constructs for skin replacement. Therefore, cultured dermal substitutes can be prepared by the production of a scaffold for the dermal component and seeding it with skin fibroblasts, capable of secreting many growth factors as well as extracellular matrix (ECM) components to fill the gaps created by the pores in the material.^2^–^5^ Subsequently, keratinocytes can be seeded upon the scaffold after appropriate dermal maturation.

## SILK: STRUCTURE AND PROPERTIES

**Figure 4:**
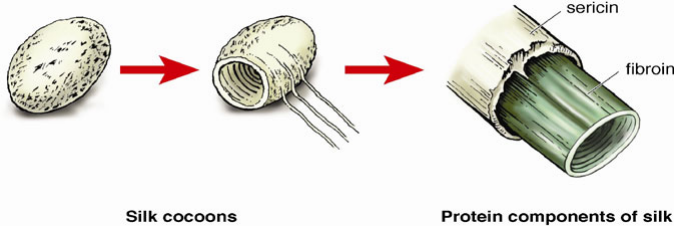
Structural components of silk.

Silks are well-known natural fibers produced by a variety of silkworm insects and spiders including, *Bombyx mori*, which is one of the most widely studied sources.^6^ Silk fibers are composed of 2 types of proteinous polymers (Figure 4):

1. Sericin, an antigenic gum-like protein that forms the outer rubbery hydrophilic coating, and
2. Fibroin, the inner-core protein filaments consisting of hydrophobic amino acids, glycine and alanine repeat sequences, which accounts for up to 90% of the total molecular weight.^7^,^8^

For decades, silk threads were used as surgical sutures until sensitization to sericin, demonstrated by type I allergic responses (asthma and upregulated levels of IgEs), were reported in patients, undergoing repeated surgical procedures.^9^–^12^ Therefore, sericin-removal is an essential step before silks can be used clinically; thus recently, silk fibroin (SF) has been increasingly investigated as a promising biomaterial for new biomedical applications.

Structurally, SF is characterized by heavy- and light-chain polypetides arranged into highly organized β-sheet crystal regions through hydrogen bonding as well as semicrystalline regions that together are responsible for the elasticity and tensile strength.^13^–^17^ Furthermore, silk fibers have greater elasticity than fibers of comparable tensile integrity^18^; for example, the elasticity of dragline silk is 4 to 7 times higher than that of synthetic high-tenacity fibers like Kevlar 49. In addition, silks are thermally stable up to approximately ˜250°C, allowing processing over a vast range of temperatures.^19^

## APPLICATION OF SF AS A BIOMATERIAL SCAFFOLD

Although silk has been used commercially for centuries, in textiles production and in many clinical applications, only recently the use of solubilized SF has been explored as a biomaterial scaffold for cell culture and TE.^20^ The biomaterial scaffold/matrix plays a key role in transducing environmental cues to cells seeded within it, acting in essence as a translator between the local environment and the developing tissue (neotissue), hence aiding the development of biologically viable functional tissue.^18^ The scaffold should essentially be designed by mimicking the structure and function of native ECM proteins, which provide mechanical support and regulate cell activities.^21^

It should support cell attachment and migration as well as guide cell differentiation and function. Furthermore, key criteria include biocompatibility and biodegradability, with nontoxic and noninflammatory degradation products during replacement in vivo by cellular ECM components.^22^ Many natural and synthetic polymers have been considered for biomaterial scaffolds; however, the challenging combination of biocompatibility, biodegradability, controllable porosity, stability for an extended time-period during neotissue growth, and processibility into porous matrixes often limit the utility of most polymers.^23^

A number of studies have demonstrated that upon sericin removal, regenerated SF has good biocompatibility,^18^,^24^–^26^ heamocompatibility,^27^ oxygen and water permeability^7^,^28^ as well as minimal inflammatory reaction. Separate studies have found that regenerated SF films prepared by dissolving silkworm cocoon fibers in 9–9.5 M lithium bromide supported the attachment and proliferation of both human and animal cell lines.^29^,^30^

Collectively, these studies have recognized that SF offers versatility in biomaterial scaffold/matrix design for use in tissue regeneration of bone, cartilage, blood vessels, ligaments, and tendons, in which mechanical performance and biological interactions are major factors for success. Furthermore, for ease of utility, SF can be processed into films, fibers, hydrogels, and meshes as shown by Min et al in 2004.^21^ A large number of fabrication techniques have been applied to process 3-dimensional polymeric scaffolds of high porosity and surface area. These include electrospinning, solvent casting/particulate leaching, emulsion freeze-drying, thermally induced phase separation, and gas foaming.

## NONWOVEN SF NANO-/MICROFIBROUS MEMBRANES

Recently, nonwoven SF membranes fabricated by electrospinning have gained attention due to the ability to produce polymer nanofibers with diameters in the range of several micrometers down to tens of nanometers.^21^ Researchers have investigated the effects of nonwoven SF microfibrous nets on the culture of a wide variety of human cell lines including osteoblasts, fibroblasts, keratinocytes, and endothelial cells. These studies have shown that these microfibrous nets support the adhesion, proliferation, and cell-cell interactions.^31^ In addition, nonwoven SF nanofibrous mats were also found to support attachment, spreading and proliferation of human bone marrow stromal cells, keratinocytes, and fibroblasts in vitro.^21^,^22^

The biocompatibility of nonwoven microfibrous membranes has been shown to be composed of partially dissolved native SF fibers.^32^ There were no infiltration of the lympocytes present in the tissue even after 6 months of subcutaneous implantation, which indicates a good biocompatibility. In addition, the implanted SF membranes were integrated with the surrounding tissue within 6 months and no obvious degradation observed. Previous in vivo studies have demonstrated SF-based membranes as promising materials for skin regeneration.^25^,^32^

## SF-BASED SCAFFOLDS AND STEM CELL-BASED TE

When considering stem cell-based TE, a reliable cell source that responds appropriately in terms of morphology, proliferation, and tissue-specific differentiation to the biomaterial scaffold is of paramount importance. Although embryonic stem cells are capable of differentiating into cell types of all different tissue lineages, a lack of understanding and control of differentiation, as well as ethical and legal boundaries, limit their use in TE. In contrast, adult stem cells with limited differentiation ability are an appealing alternative. Mesenchymal stem cells are one such example, which can be isolated from various adult tissue including adipose tissue,^33^ articular cartilage,^34^ bone marrow,^35^,^36^ and synovium.^37^ Mesenchymal stem cells and SF-based scaffolds have been extensively studied in ligament, cartilage, and bone. However, there are currently limited published reports on their use in skin, providing an area if interesting for further research.

## DISCUSSION

Human skin is considered as one of the most important organs of the body, providing a multitude of structural and functional benefits, ensuring perfect homeostasis.^38^ Since tissue loss at the skin level is a common occurrence due to a multitude of events such as lacerations, cutaneous disease, neoplasia, infection, burns and other trauma, adequate cutaneous constructs that could act as effective skin replacements, capable of mimicking native skin are highly desirable.^39^

There are currently 2 different types of commercially available bilayered cutaneous constructs.^40^ However, they are at present unable to fulfill all the structural and functional properties of native skin; thus, the scope for engineering a novel cutaneous construct remains.^41^ The development of innovative skin replacements has previously been along the 3 approaches of TE, although the scaffold approach commonly created from either modified collagens or resorbable polymers is most frequently used.^42^

In vitro investigation has shown that several types of human cell lines, including dermal fibroblasts, epidermal keratinocytes, and endothelial cells, can be successfully cultured on SF scaffolds in various forms; therefore, cell attachment, proliferation, and differentiation can be studied accordingly.^21^,^22^ In addition, after in vivo implantation subcutaneously, the SF implants were shown to integrate well with the surrounding tissue while no host immunologic response was reported.^25^,^32^ Hence, the suitability of SF as a novel type of biomaterial scaffold to be used for TE has been clearly demonstrated.

This encouraging breakthrough was attributed to the innovative characteristics of SF, which includes the following:

• Good interaction with human cells in vitro that support cell-specific needs.^21^,^22^
• Compatibility in vivo following implantation without evoking a foreign body response and therefore being capable of fully integrating into the surrounding host tissue.^25^,^32^
• It can be processed into aqueous solutions for subsequent formation of films as well as other material formats.^21^
• Degradability at a controlled rate both in vitro and in vivo, which is of particular importance with regards to biodegradable scaffolds where slow neotissue growth is most desirable.^18^

SF also has novel mechanical properties that are capable of rivaling many natural or synthetic high-performance fibers.^13^–^18^ Consequently, SF has been established as a highly promising biomaterial for its surface morphology, superior structural and mechanical properties, in association with good biocompatibility and biodegradability, thus proving to be a suitable scaffold for TE applications and subsequent development of novel cutaneous constructs.

## CONCLUSIONS

Tissue-engineered skin has advanced from the initial cultured autologous epidermal sheets to more complex bilayered cutaneous constructs capable of mimicking selective structures and functions of native skin. Silk fibroin, 1 of 2 proteins found in naturally occurring silk, has been gaining momentum as a promising material for biomedical application due to its ability to be biocompatible with the host immune system, as well as support cell attachment, proliferation, and differentiation, which are key components for TE. Consequently, the potential advantages of SF as a biomaterial scaffold are substantial, with the provision of further possibilities in cell culture and TE, implying that adequate cutaneous constructs are a realistic prospect in the not too distant future.

## Figures and Tables

**Table 1:** Current cutaneous substitutes—divided into epidermal constructs, dermal constructs, and complete bilayered (both epidermal and dermal) constructs

**Type of construct**	**Trade name**	**Prominent cell type**	**Layers**
Epidermal construct	Laserskin^TM^	Keratinocytes	Cultured autograft sheets
Dermal constructs	AlloDerm^TM^	Acellular	De-epithelialized cadaver dermis
	Integra^TM^	Acellular	1. Silicone sheet
			2. Collagen
			3. Glycosaminoglycan
	Dermagraft^TM^	Fibroblasts	Polyglycolic acid or Poly(glactin)-910 scaffold seeded with neonatal fibroblasts
	TransCyte^TM^	Fibroblasts	1. Silicone sheet
			2. Nylon mesh
			3. Collagen produced by neonatal fibroblasts
Complete bilayered constructs	Apligraf^TM^	Keratinocytes and fibroblasts	1. Allogeneic keratinocytes
			2. Allogeneic fibroblasts
			3. Bovine collagen gel
	Academia^TM^	Keratinocytes and fibroblasts	1. Allogeneic keratinocytes
			2. Allogeneic fibroblasts
			3. Gylcosaminoglycan
			4. Bovine collagen scaffold
	OrCel^TM^	Keratinocytes and fibroblasts	1. Allogeneic keratinocytes
			2. Allogeneic fibroblasts
			3. Nonporous collagen gel
			4. Bovine collagen scaffold

## References

[1] Lee SB, Kim YH, Chong MS (2005). Study of gelatin-containing artificial skin V: fabrication of gelatin scaffolds using a salt-leaching method. Biomaterials.

[2] Naughton GK (2000). Principles of Tissue Engineering.

[3] Cooper ML, Hansbrough JF, Spielvogel RL, Cohen R, Bartel RL, Naughton G (1991). In vivo optimization of a living dermal substitute employing cultured human fibroblasts on a biodegradable polyglycolic acid or polyglactin mesh. Biomaterials.

[4] Mansbridge J, Liu R, Patch R (1998). Three-dimensional fibroblast culture implant for the treatment of diabetic foot ulcers: metabolic activity and therapeutic range. Tissue Eng.

[5] Naughton GK, Mansbridge JN (1999). Human-based tissue-engineered implants for plastic and reconstructive surgery. Clin Plast Surg.

[6] Jin HJ, Kaplan DL (2003). Mechanism of silk processing in insects and spiders. Nature.

[7] Minoura N, Tsukada M, Nagura M (1990). Physico-chemical properties of silk fibroin membrane as a biomaterial. Biomaterials.

[8] Minoura N, Aiba SI, Gotoh Y, Tsukada M, Imai Y (1995). Attachment and growth of cultured fibroblast cells on silk protein matrices. J Biomed Mater Res.

[9] Soong HK, Kenyon KR (1984). Adverse reactions to virgin silk sutures in cataract surgery. Ophthalmology.

[10] Wen C, Ye S, Zhou L, Yu Y (1990). Silk-induced asthma in children: a report of 64 cases. Ann Allergy.

[11] Kurosaki S, Otsuka H, Kunitomo M, Koyama M, Pawankar R, Matumoto K (1999). Fibroin allergy. IgE mediated hypersensitivity to silk suture materials. Nippon Ika Daigaku zasshi.

[12] Rossitch E, Bullard DE, Oakes WJ (1987). Delayed foreign-body reaction to silk sutures in pediatric neurosurgical patients. Childs Nerv Syst.

[13] Simmons A, Ray E, Jelinski LW (1994). Solid-state 13C NMR of *Nephila clavipes* dragline silk establishes structure and identity of crystalline regions. Macromolecules.

[14] Simmons A, Michal CA, Jelinski LW (1996). Molecular orientation and two-component nature of the crystalline fraction of spider dragline silk. Science.

[15] Vollrath F, Knight DP (2001). Liquid crystalline spinning of spider silk. Nature.

[16] Vollrath F (2000). Strength and structure of spiders' silks. J Biotechnol.

[17] Vollrath F (2005). Spiders' webs. Curr Biol.

[18] Altman GH, Diaz F, Jakuba C (2003). Silk-based biomaterials. Biomaterials.

[19] Wong Po, Foo C, Kaplan DL (2002). Genetic engineering of fibrous proteins: spider dragline silk and collagen. Adv Drug Deliv Rev.

[20] Yamada H, Nakao H, Takasu Y, Tsubouchi K (2001). Preparation of undergraded native molecular fibroin solution from silkworm cocoons. Mater Sci Eng C.

[21] Min BM, Lee G, Kim SH, Nam YS, Lee TS, Park WH (2004). Electrospinning of silk fibroin nanofibers and its effect on the adhesion and spreading of normal human keratinocytes and fibroblasts in vitro. Biomaterials.

[22] Jin HJ, Chen J, Karageorgiou V, Altman GH, Kaplan DL (2004). Human bone marrow stromal cell responses on electrospun silk fibroin mats. Biomaterials.

[23] Nazarov R, Jin HJ, Kaplan DL (2004). Porous 3-D scaffolds from regenerated silk fibroin. Biomacromolecules.

[24] Santin M, Motta A, Freddi G, Cannas M (1999). In vitro evaluation of the inflammatory potential of the silk fibroin. J Biomed Mater Res.

[25] Sugihara A, Sugiura K, Morita H (2000). Promotive effects of a silk film on epidermal recovery from full-thickness skin wounds (44552). Exp Biol Med.

[26] Meinel L, Hofmann S, Karageorgiou V (2005). The inflammatory responses to silk films in vitro and in vivo. Biomaterials.

[27] Sakabe H, Ito H, Miyamoto T, Noishiki Y, Ha WS (1989). In vivo blood compatibility of regenerated silk fibroin. Sen-i Gakkaishi.

[28] Minoura N, Tsukada M, Nagura M (1990). Fine structure and oxygen permeability of silk fibroin membrane treated with methanol. Polymer.

[29] Gotoh Y, Tsukada M, Minoura N (1998). Effect of the chemical modification of the arginyl residue in *Bombyx mori* silk fibroin on the attachment and growth of fibroblast cells. J Biomed Mater Res.

[30] Inouye K, Kurokawa M, Nishikawa S, Tsukada M (1998). Use of *Bombyx mori* silk fibroin as a substratum for cultivation of animal cells. J Biochem Biophys Methods.

[31] Unger RE, Wolf M, Peters K, Motta A, Migliaresi C, Kirkpatrick CJ (2004). Growth of human cells on a non-woven silk fibroin net: a potential for use in tissue engineering. Biomaterials.

[32] Dal Pra I, Freddi G, Minic J, Chiarini A, Armato U (2005). De novo engineering of reticular connective tissue in vivo by silk fibroin nonwoven materials. Biomaterials.

[33] Nathan S, De SD, Thambyah A, Fen C, Goh J, Lee EH (2003). Cell-based therapy in the repair of osteochondral defects: a novel use for adipose tissue. Tissue Eng.

[34] Alsalameh S, Amin R, Gemba T, Lotz M (2004). Identification of mesenchymal progenitor cells in normal and osteoarthritic human articular cartilage. Arthritis Rheum.

[35] Caplan AI (1991). Mesenchymal stem cells. J Orthop Res.

[36] Pittenger MF, Mackay AM, Beck SC (1999). Multilineage potential of adult human mesenchymal stem cells. Science.

[37] De Bari C, Dell'Accio F, Tylzanowski P, Luyten FP (2001). Multipotent mesenchymal stem cells from adult human synovial membrane. Arthritis Rheum.

[38] Haake AR, Holbrook K, Wolff K, Goldsmith LA, Katz SI, Gilchrest BA, Paller A, Leffell DJ (2007). Development and structure of skin. Fitzpatrick's Dermatology in General Medicine.

[39] Ehrenreich M, Ruszczak Z (2006). Update on tissue-engineered biological dressings. Tissue Eng.

[40] Badiavas EV, Paquette D, Carson P, Falanga V (2002). Human chronic wounds treated with bioengineered skin: histologic evidence of host-graft interactions. J Am Acad Dermatol.

[41] Tremblay PL, Hudon V, Berthod F, Germain L, Auger FA (2005). Inosculation of tissue-engineered capillaries with the host's vasculature in a reconstructed skin transplanted on mice. Am J Transplant.

[42] Auger FA, Berthod F, Moulin V, Pouliot R, Germain L (2004). Tissue-engineered skin substitutes: from in vitro constructs to in vivo applications. Biotechnol Appl Biochem.

